# Unpredictable chronic mild stress differentially impairs social and contextual discrimination learning in two inbred mouse strains

**DOI:** 10.1371/journal.pone.0188537

**Published:** 2017-11-22

**Authors:** Michiel van Boxelaere, Jason Clements, Patrick Callaerts, Rudi D’Hooge, Zsuzsanna Callaerts-Vegh

**Affiliations:** 1 Laboratory of Biological Psychology, KU Leuven, Leuven, Belgium; 2 Laboratory of Behavioral and Developmental Genetics, KU Leuven, Leuven, Belgium; 3 Leuven Research Institute for Neuroscience & Disease (LIND), Leuven, Belgium; 4 mINT Mouse Behavioral Core Facility, KULeuven, Leuven, Belgium; Klinikum der Johann Wolfgang Goethe-Universitat Frankfurt, GERMANY

## Abstract

Alterations in the social and cognitive domain are considered important indicators for increased disability in many stress-related disorders. Similar impairments have been observed in rodents chronically exposed to stress, mimicking potential endophenotypes of stress-related psychopathologies such as major depression disorder (MDD), anxiety, conduct disorder, and posttraumatic stress disorder (PTSD). Data from numerous studies suggest that deficient plasticity mechanisms in hippocampus (HC) and prefrontal cortex (PFC) might underlie these social and cognitive deficits. Specifically, stress-induced deficiencies in neural plasticity have been associated with a hypodopaminergic state and reduced neural plasticity persistence. Here we assessed the effects of unpredictable chronic mild stress (UCMS) on exploratory, social and cognitive behavior of females of two inbred mouse strains (C57BL/6J and DBA/2J) that differ in their dopaminergic profile. Exposure to chronic stress resulted in impaired circadian rhythmicity, sociability and social cognition in both inbred strains, but differentially affected activity patterns and contextual discrimination performance. These stress-induced behavioral impairments were accompanied by reduced expression levels of brain derived neurotrophic factor (BDNF) in the prefrontal cortex. The strain-specific cognitive impairment was coexistent with enhanced plasma corticosterone levels and reduced expression of genes related to dopamine signaling in hippocampus. These results underline the importance of assessing different strains with multiple test batteries to elucidate the neural and genetic basis of social and cognitive impairments related to chronic stress.

## Introduction

Chronic stressful life events are a major risk factor in the development and maintenance of many psychopathologies [1]. Stress-exposure often perturbs one`s physiological and psychological functioning leading to behavioral dysfunctions in the affective, social and cognitive domain [2,3]. Among other well-defined symptoms, social and cognitive disturbances are considered to be a major contributor to the burden of disease in patients suffering from stress-related disorders such as major depression disorder (MDD), anxiety, Cushing`s syndrome, and posttraumatic stress disorder (PTSD) [4]. Impairments in attention [5], processing speed [6], executive functioning [7], learning and memory [8,9] have been widely reported in these patients. However, the neuropathological mechanisms underlying these behavioural dysfunctions are still not fully understood. Different animal models have been established and used to investigate what mediates these stress-induced anomalies [10–12].

In accordance with human studies, animals subjected to chronic stress mimic many cognitive impairments in different learning and memory protocols such as Morris water maze [13], passive avoidance [14] and radial arm maze [15]. Potentially underlying these behavioural anomalies, chronic stress has been shown to have detrimental effects on HC and PFC structure and function [16–19]. In these particular brain structures, expression of genes involved in neuronal plasticity [20] and dopamine-dependent memory persistence have been shown to be dysregulated in patients and animal models of PTSD, Cushing`s syndrome and other stress-related psychopathologies [17,21–25]. Brain derived neurotrophic factor (BDNF) is involved in the development, growth and differentiation of novel neurons as well as in the survival of existing neurons [26]. Long-term exposure to stress leads to decreased BDNF expression in HC [27] and PFC [28], which has been proposed to be related to cognitive impairments observed in chronically stressed mice [29]. Accordingly, post-mortem studies revealed a significant reduction of BDNF in PFC and HC of patients suffering from stress-related mood disorders [30].

Among other neural populations, ventral tegmental area (VTA) dopamine (DA) neurons have been shown to regulate the expression of proteins, such as BDNF, necessary for lasting neuronal plasticity in HC [31,32] and PFC [33]. DA release in NAc, PFC and HC is considered to be essential in motivational vigour [34], social behaviour [35] and long-term memory persistence [36,37]. In humans for example, genetic predisposition for increased DA availability is a putative resilience factor for negative emotionality and depression [38], whereas microstructural abnormalities in midbrain and subcortical regions (including VTA) have been observed in depressed patients [39]. In animal models, DAergic neurotransmission has been shown to be directly involved in mediating stress responses [40,41], depression-like behaviour [42], as well as in determining the balance between susceptibility versus resilience to stress-induced behavioural abnormalities [43]. However, the extent to which DAergic neurotransmission is involved in stress-induced cognitive decline remains unclear.

In this study we investigated the effects of unpredictable chronic mild stress (UCMS) on a battery of exploration, social and cognitive tests in two inbred mouse strains. UCMS is a well-established rodent model for stress-related psychopathologies, displaying changes at the molecular, anatomical, and behavioral level comparable to clinical observations [10,44–49]. The inbred strains C57BL/6J and DBA/2J are frequently used and have been shown to differ in their reaction to stressful manipulations [50], social and cognitive behavior [51,52], HC and PFC synaptic plasticity [53,54], as well as in their DAergic profile [55]. The goal of these experiments was to investigate the impact of genetic background on stress-induced social and cognitive impairments and how this correlates with the expression levels in the HC and PFC of genes implicated in dopaminergic neurotransmission and neuronal plasticity.

## Materials and methods

### Animals

**Fig 1:**
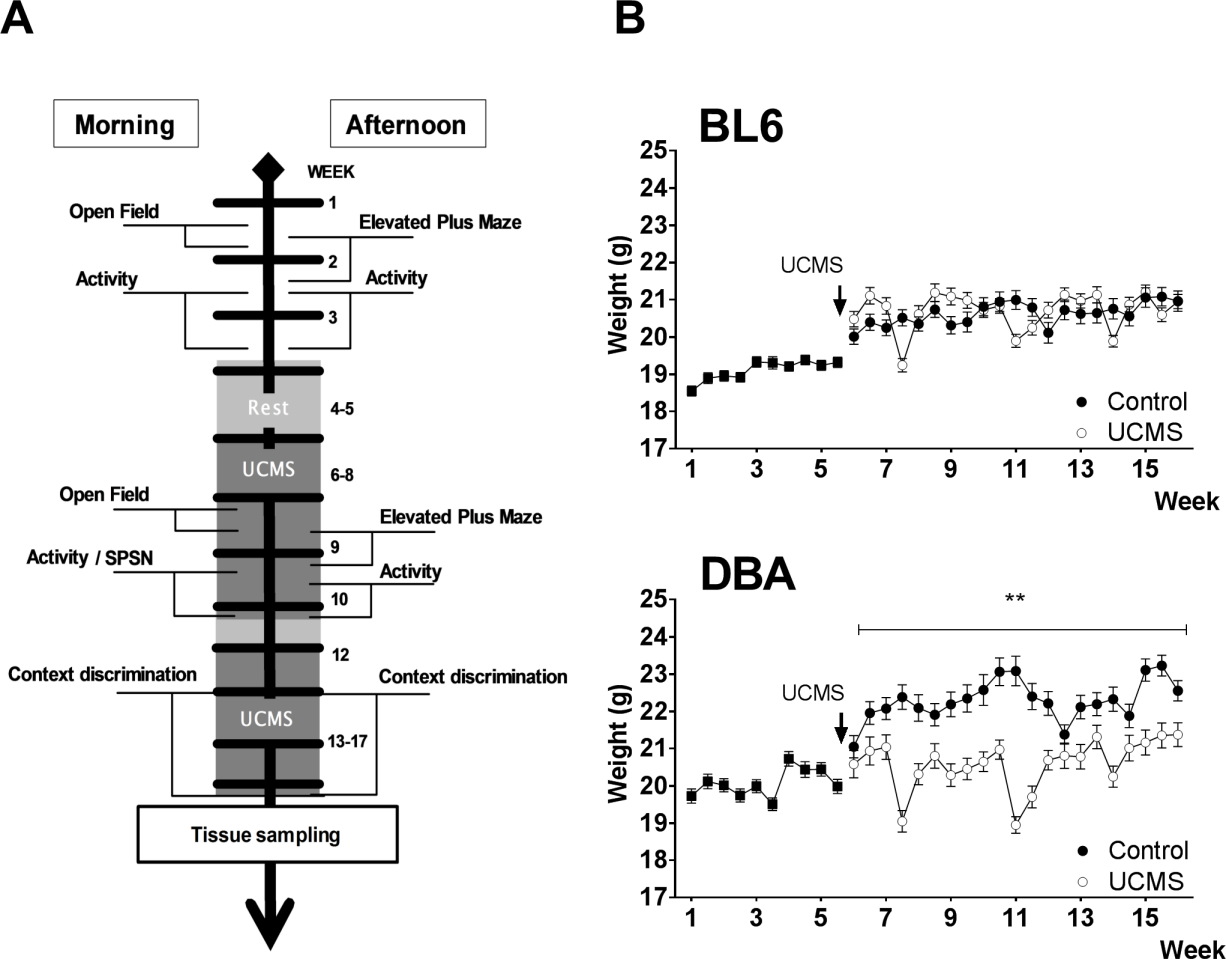
Schematic representation of experimental design. (A) and effects of UCMS on body weight (B), measured over the complete behavioral testing period. Data are presented as mean +/- SEM. ** p < 0.01.

Subjects were 30 female mice from the DBA/2J inbred strain (DBA) and 30 female mice from the C57BL/6J inbred strain (BL6) (Elevage Janvier, Le Genest Saint Isle, France). Upon arrival mice were 8 weeks old. Mice were group housed (7/8 per cage) in standard animal cages throughout the entire experiment and kept under temperature and humidity controlled conditions (12h/12h light-dark cycle with lights on at 8:00 a.m., 22°C). Food and water were available ad libitum. Following baseline testing (week 1–3), mice were randomly assigned to one of two groups: controls and exposure to UCMS. Animals in the control group were provided cage enrichment (carboard rolls, nesting material), whereas UCMS-exposed mice were not. Body weight was closely monitored (Fig 1B). All behavioral testing was performed during the light phase of the activity cycle, with the exception of the 23h-activity test. For the first series of behavioral tests (open field, activity, elevated plus maze and sociability/preference for social novelty test), 60 animals (15/group) were tested. In contextual discrimination, 48 animals (12 /group) were tested. One mouse died during discrimination training and was excluded from analysis.

All experimental procedures comply with international guidelines on the ethical use of animals. All experiments have been reviewed and approved by the animal ethical committee of the University of Leuven, Belgium (project number: 199/2015). All efforts were made to minimize animal suffering where possible.

### Design of the experiment

Mice from each strain were randomly distributed across two groups (CTRL and UCMS). Behavioral tests were conducted either in the morning (8 a.m.- 1 p.m.) or in the afternoon (2 p.m.-7 p.m.) to limit between-group circadian variation (Fig 1A). Upon arrival, mice were given a 7-day adaptation period to habituate to housing and handling conditions. To set a behavioral baseline, mice were tested over a three-week period in the following sequence: open field test, elevated plus-maze, 23h-activity test. Following a two-week rest period (week 4–5), the unpredictable chronic mild stress procedure started for four weeks and was continued during behavioral testing (Table 1). Exploratory and sociability tests (week 8–10) were conducted in the following order: open field test, elevated plus-maze, 23h-activity test and SPSN test. In week 10, UCMS exposure was paused and three mice/group were sacrificed for corticosterone (CORT) analysis. One week later (week 11), UCMS was resumed for 10 days. Followed by a contextual discrimination threat conditioning task (week 13–17). After completion of this contextual discrimination task (week 17), blood and brain samples were collected over a three-day period. Control conditions were tested in parallel with the UCMS group and continued to be group housed under normal lab conditions, with cage enrichment and minimal handling. Sample collections were equally distributed across groups per day to avoid timing related confounders. The estrous cycle was not checked during the experiment.

**Table 1:** Unpredictable chronic mild stress procedure.

	Monday	Tuesday	Wednesday	Thursday	Friday	Saturday	Sunday
**Morning**	Cold exposure		Light on/off	**Open field**	**Open field**	**Open field**	
**Afternoon**		Predator smell	Damp sawdust	Tilted Cage	Vinegar exposure		Damp sawdust
**Overnight**	Bedding removed	Food deprivation				Overnight illumination	Overnight illumination

Example of weekly schedule for unpredictable chronic mild stress procedure during open field test

### Unpredictable chronic mild stress procedure

Mice were repeatedly subjected to mild stressors such as: exposure to wet sawdust, placement in a cage without bedding material, randomly switching lights on and off (on average every 20 min), bright light exposure, cage tilting (45°), exposure to vinegar (5% acetic acid in water), overnight food removal, 45 min cold exposure (4°C), overnight illumination and exposure to predator (rat) smell. Initially, mice were exposed to two of these stressors per day for a four week period in a semi-random order (see Fig 1A). After a two-week resting period, mice again were exposed to on average two stressors per day for 10 consecutive days. During testing days, mice were subjected to only one stressor (see Table 1). No stressors were applied 12 h prior to behavioral tests. All stress manipulations were performed in a room different from the housing room, with the exception of cage tilting and overnight food removal.

### Behavioral testing

#### Exploratory behavior

Locomotor activity and exploratory behavior was measured in the open field test as previously described [56]. After a 30 min dark-adaption period, mice were placed in a brightly lit open field area (50 x 50 cm^2^). Following 60 s habituation, exploratory behavior was recorded for 10 min using an automated video tracking system (ANY-maze™ Video Tracking, Stoelting Co. IL, USA). Variables analyzed were path length and time spent in the center of the open field arena.

Anxiety-related exploration was measured in the elevated plus-maze [56]. Mice were placed in the center of a plus-shaped maze, consisting two open arms (5 cm wide) without walls and two arms closed by side arms. Anxiety-related exploration was recorded for 11 min (1 min habituation and 10 min recording) by five IR photo beams connected to a computerized activity logger. There is one IR photo beam at the entry of each arm. One IR beam records the relative time spent in the open arm.

To measure circadian cage activity [57], mice were placed individually in transparent cages (26.7 cm x 20.7 cm). These cages were placed between three IR photo beams connected to a computerized activity logger. Activity was registered as the number of beam crossings for each 30 min interval, during a 23 h recording period. Following a 15 min habituation, registration of beam crossings started at 6:30 pm during the pre-UCMS activity test and at 4 pm during the post-UCMS activity test, with lights being switched off at 20 h (12h on/off cycle). Cage activity during the dark phase of the recording period was analyzed.

#### Sociability/preference for social novelty

Sociability/preference for social novelty (SPSN) test setup was first described by Nadler et al. [58] and modified in our lab [57,59]. The setup consisted of an enclosed rectangular transparent Plexiglas box with an opaque floor (*w x d x h*: 94 x 26 x 30 cm), divided into three chambers. The central chamber (42 x 26 cm) was connected to a left and right chamber (26 x 26 cm) via openings (6 x 8 cm) in division walls between chambers. Left and right chamber contained cylindrical wire cups (*height x diameter*: 11 x 10 cm) that could contain a stranger mouse. Access to the chambers was controlled by manually operated guillotine doors. Two cameras were placed 60 cm above the setup to track animal movement. Movement pathways and approach behavior were recorded and analyzed using ANY-maze™ Video Tracking System software (Stoelting Co. IL, USA).

The SPSN test consisted of three consecutive stages: acclimation stage, sociability stage, and preference for social novelty stage. During an acclimation stage, a test mouse was placed in the bottom right corner of the central chamber with both guillotine doors closed. During this stage, empty wire cups in the left and right chamber were visible from the central chamber. Mice were allowed to explore the central chamber freely for 5 min. In the sociability stage, a stranger mouse (STR1) was placed in one of the wire cups, the position (left or right) was determined randomly, while the other wire cup remained empty. Recording was started and the guillotine doors were opened allowing the test animal to explore all three chambers for 10 min. After 10 min, the animal was again placed in the central chamber with closed guillotine doors and the next stage (preference for social novelty) was initiated. A novel stranger mouse (STR2) was placed in the empty cup. Recording started and both doors again opened to allow free access to both sides for 10 min. Between test animals, stranger mice were replaced and the setup was cleaned thoroughly using water and paper tissues. At the end of each test day, the setup was cleaned with a 70% ethanol solution. Stranger mice were 7-month old, group-housed (5 per cage) female C57BL/6J mice specifically used for SPSN or social exploration tests. Each stranger mouse served once as STR1 and once as STR2 per testing day. STR1 and STR2 were always picked from different housing cages.

#### Contextual discrimination threat conditioning

Contextual discrimination threat conditioning between similar contexts was conducted in a Panlab Startle & Fear Combined System (Panlab, S/L/, Cornellà, Spain) using a protocol based on Nakashiba and colleagues [60]. Two identical conditioning boxes (25 x 25 x 25 cm) with stainless steel grid floors to deliver shocks were located in sound-attenuating cubicles. Animal movement was monitored by motion-sensitive floors connected to an interfaced computer using Panlab Freezing v1.2.0 software. The degree of motion could range from 0 to 100. Freezing was counted if registered movement remained below a threshold of 2.5 (arbitrary unit) for at least 1 s [61]. Animals were threat conditioned in context A, and freezing behavior in an alternate context (B) was recorded as a measure of discrimination learning. Context B was identical to A, except for an inserted A-frame roof made from black cardboard. Animals were transported to a holding area in their home cages and left undisturbed for 30 min. After conditioning mice were kept separately until its cohabitants had also been tested. Contextual discrimination training consisted of four phases: contextual threat acquisition, generalization test, discrimination training and a second generalization test.

In contextual threat acquisition (day 1–3), mice were placed in context A and after 3 min exploration a foot shock (2 sec; 0.5 mA) was delivered. One minute later, mice were removed from the testing box and placed in a housing cage. Freezing was measured during the 3 min interval preceding the shock. To determine the specificity of contextual threat conditioning, freezing behavior in contexts A and B was recorded during the test for generalization (day 4–5). On day 4, mice were placed in context A or B without shocks for 3 min, then removed and placed in a housing cage. 120–150 min later, mice were placed in the other context (A or B). The order of contexts was counterbalanced. Half of the animals were placed in context A first, whereas the other half was firstly placed in context B. On day 5, the same procedure was used and the order of context presentation was switched. Freezing was measured during a 3 min interval.

During discrimination training (day 6–27), mice were trained daily in each context (A or B) once, following a double alternation procedure: on day 6, A → B; day 7, B → A: day 8, B→ A; day 9, A → B; etc. In context A, mice received a foot shock (2 sec; 0.5 mA) after 3 min exploration time, and were removed 60 s later. In context B, the same test duration was applied (4 min and 2 s) but without foot shocks. Freezing was measured during the first 3 min of context exposure. Data are collapsed into two consecutive day training blocks, containing both alternations. Another test for context generalization was performed on days 28–29 (identical to day 4–5).

### Biomarkers

#### CORT (corticosterone) analysis

Mice (3 and 5 per group) were deeply anaesthetized with nembutal (i.p. 60 mg/kg) and locally with xylocaine (2%, 0.005 ml peri-orbital) before blood was collected (retro-orbitally) for serum corticosterone quantification. Samples were collected between 9 and 12 a.m., using heparin coated blood collection capillaries. Blood samples were quickly centrifuged at high speed (14,000 g) for 5 min to collect plasma, which was stored separately at -80°C until quantification. Plasma corticosterone concentrations were measured using a commercially available RIA kit (IDS Ltd., Bolden, UK).

#### qRT-PCR

After blood collection, mice were killed by decapitation and brains were removed, dissected on ice and flash frozen for further mRNA analysis. Total mRNA was isolated from hippocampi and medial prefrontal cortex using mRNA isolation kit (miRVana™, Ambion™, Thermo Fischer Scientific). Briefly, after homogenization in lysis buffer, RNA was extracted in acidic phenol-chloroform solution and isolated over glass-fiber filters. After washing steps, total RNA was eluted from the filters and stored at −80°C until further processing. Total RNA concentration was quantified using the NanoDrop^®^method (ND-1000 spectrophotometer, Thermo Scientific, USA). Quantitative real-time polymerase chain reaction (qRT-PCR) was carried out using fluorescent 6-FAM probes (6-carboxyfluorescein, Applied Biosystems™, USA). RNA was reverse-transcribed to cDNA using primers specific to each mRNA gene of interest on Applied Biosystem's GeneAmp PCR System 9700. qRT-PCR was carried out on a StepOnePlus^TM^ PCR machine (Applied Biosystems, UK). Samples were heated to 95°C for 10 min, and then subjected to 40 cycles of amplification by melting at 95°C and annealing at 60°C for 1 min. Each biological replicate was run in technical replicates with 1.33 μl cDNA per reaction. To check for amplicon contamination, each run also contained template free controls for each probe used. The following primer pairs were used: *bdnf*, forward: 5’-TACCTGGATGCCGCAAACAT-3’, reverse: 5’-TGCTGTGACCCACTCGCTAAT-3’*; Ppp1r1b* (DARPP-32), forward: 5’-CCCATCACTGAAAGCTGTGC-3’, reverse: 5’-TCCCGAAGCTCCCCTAACTC-3’; *Slc6a3* (DAT), forward: 5’- ACGCTGGAGGCAGTCGAA -3’, reverse: 5’- GGGCCACCACAGAAGACATT-3’; *NR4a1* (NUR77), forward: 5’- CTGCGAAAGTTGGGGGAGT-3’, reverse: 5’-CTTGAATACAGGGCATCTCCAG-3’ and *th*, forward: 5’-TGTCACGTCCCCAAGGTTCA-3’, reverse: 5’-CTCCAATGGGTTCCCAGGTT-3’ PCR data were analyzed using the 2^−^*Δ*^Ct^ method.

#### Statistical analysis

Group means were statistically compared using Student t-tests or analyses of variance (ANOVA) for parametric data and Mann-Whitney’s U test for non-parametric data. Results of multiple trials or time points were compared using two-way repeated measures ANOVA. Post hoc comparisons were performed using Sidak multiple comparisons tests, as well as Kolmogorov-Smirnov (K-S) test for cumulative distributions. Analysis were conducted using SPSS vs. 20.0 (IBM Corp., released 2011, Armonk, NY) and GraphPad Prism 7.0 software (GraphPad Software, Inc., San Diego, CA). All statistics were performed with α = 0.05. Data are presented as mean ± SEMs.

## Results

### Body weight

When exposed to UCMS, (week 5–17), DBA animals displayed large variation in bodyweight and no overall weight gain over 12 weeks compared to non-UCMS controls [F(1;14) = 19.25, p < 0.01; Fig 1B]. In contrast, mice with C57BL/6j background, displayed similar weight gain as their controls [F(1;14) = 0.052, p = 0.822; Fig 1B].

### Exploratory behavior

**Fig 2:**
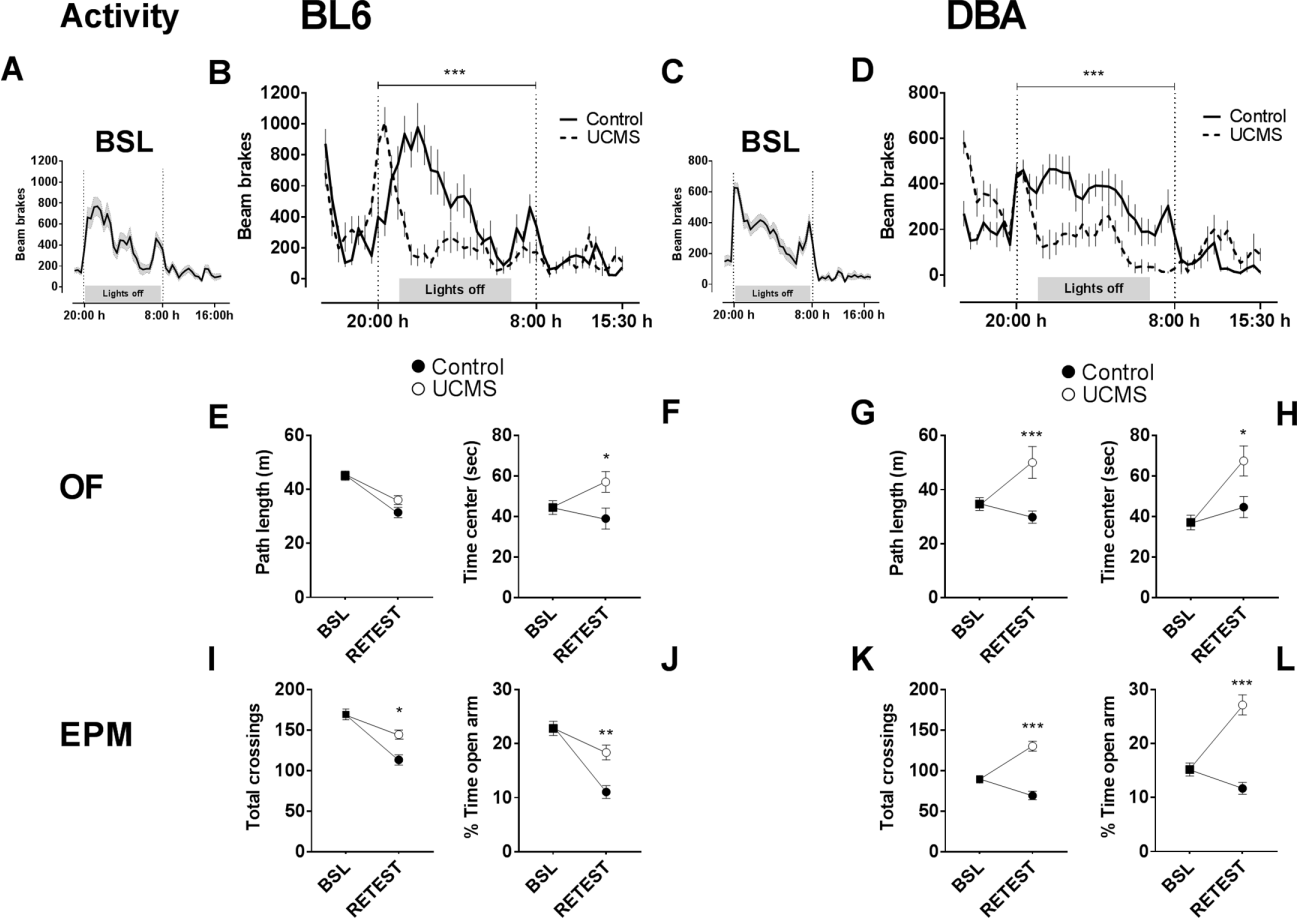
Effect of UCMS on overnight activity, exploratory and anxiety-like behavior. (A-D) In the overnight activity test, at baseline testing BL6 mice (A) were more active than DBA mice (C), but showed similar activity patterns. Groups exposed to UCMS (C, D) showed decreased overnight activity. In the open-field test, (E) BL6 mice exposed to UCMS (white circles) do not differ from CTRLS (black circles) on path length. (G) DBA exposed to UCMS showed increased distance travelled. (F) UCMS-exposed BL6 mice and (H) UCMS-exposed DBA mice spent significantly more time in the center. In the elevated plus maze, total number of arm entries were recorded as well as time spent in the open arms. (I, J) BL6 mice exposed to UCMS and (K, L) UCMS-exposed DBA mice show increased beam crossings and time spent in the open arms. Values are expressed as mean +/- SEM. *, p < 0.05; ** p < 0.01; *** p < 0.001; UCMS vs. CTRL.

In the overnight activity baseline test (Fig 2A and 2C), prior to UCMS-exposure, BL6 mice were generally more active overnight when compared to DBA mice [F(1, 58) = 13.74, p < 0.001]. UCMS exposure decreased overnight activity in BL6 mice [F(1, 28) = 14.43, p < 0.001; Fig 2B] and DBA mice [F(1, 28) = 20.97, p < 0.001; Fig 2D] in comparison to control animals.

In the open field test (Fig 2E–2H), a significant decrease of distance travelled over time was observed in BL6 mice [F(1,28) = 10.66, p = 0.003; Fig 2E], but not in DBA mice [F(1,28) = 3.304 p = 0.079; Fig 2G]. Distance travelled was increased in UCMS DBA mice [F(1,28) = 10.48, p = 0.003; Fig 2G], but not in UCMS BL6 mice [F(1,28) = 0.049, p = 0.826; Fig 2E]. Post-hoc comparisons confirmed increased distance travelled following UCMS-exposure in DBA mice (t_56_ = 3.702, p < 0.001). Furthermore, UCMS-exposure lead to altered anxiety-like behavior in both BL6 mice [F(1,28) = 5.655, p = 0.024; Fig 2F] and DBA mice [F(1,28) = 4.945, p < 0.034; Fig 2H] as measured by time spent in the center. Both BL6 mice (t_56_ = 2.69, p < 0.05) and DBA mice (t_56_ = 2.812, p < 0.05) spent more time in the center of the open field following UCMS exposure in comparison to controls.

In the elevated plus maze (Fig 2I–2L), activity from baseline was generally decreased in BL6 mice [F(1,28) = 56.6, p < 0.001; Fig 2I], not in DBA mice [F(1,28) = 3.902, p < 0.058; Fig 2K]. Both BL6 mice [F(1, 28) = 5.092, p = 0.032; Fig 2I] and DBA mice [F(1, 28) = 22.02, p < 0.001; Fig 2K] showed increased arm entries following UCMS-exposure. Moreover, UCMS-exposed animals generally spent more time in the open arm, regardless of strain [BL6; F(1,28) = 12.97, p = 0.001; Fig 2J and DBA; F(1,28) = 22.47, p < 0.001; Fig 2L].

### Sociability/preference for social novelty (SPSN)

During acclimation phase, DBA mice under UCMS regimen were more active than their controls (path length 7.79 ± 2.51 m and 18.32 ± 4.63 m for CTRL and UCMS, respectively. t_28_ = 7.738, p < 0.001). In contrast, UCMS regimen had no effect on path length in BL6 (path length 12.07 ± 3.42 m and 12.49 ± 2.43 m for CTRL and UCMS, respectively. t_28_ = 0.391, p = 0.698). This specific increase in exploratory behavior in UCMS DBA was also observed during the other phases (data not shown).

**Fig 3:**
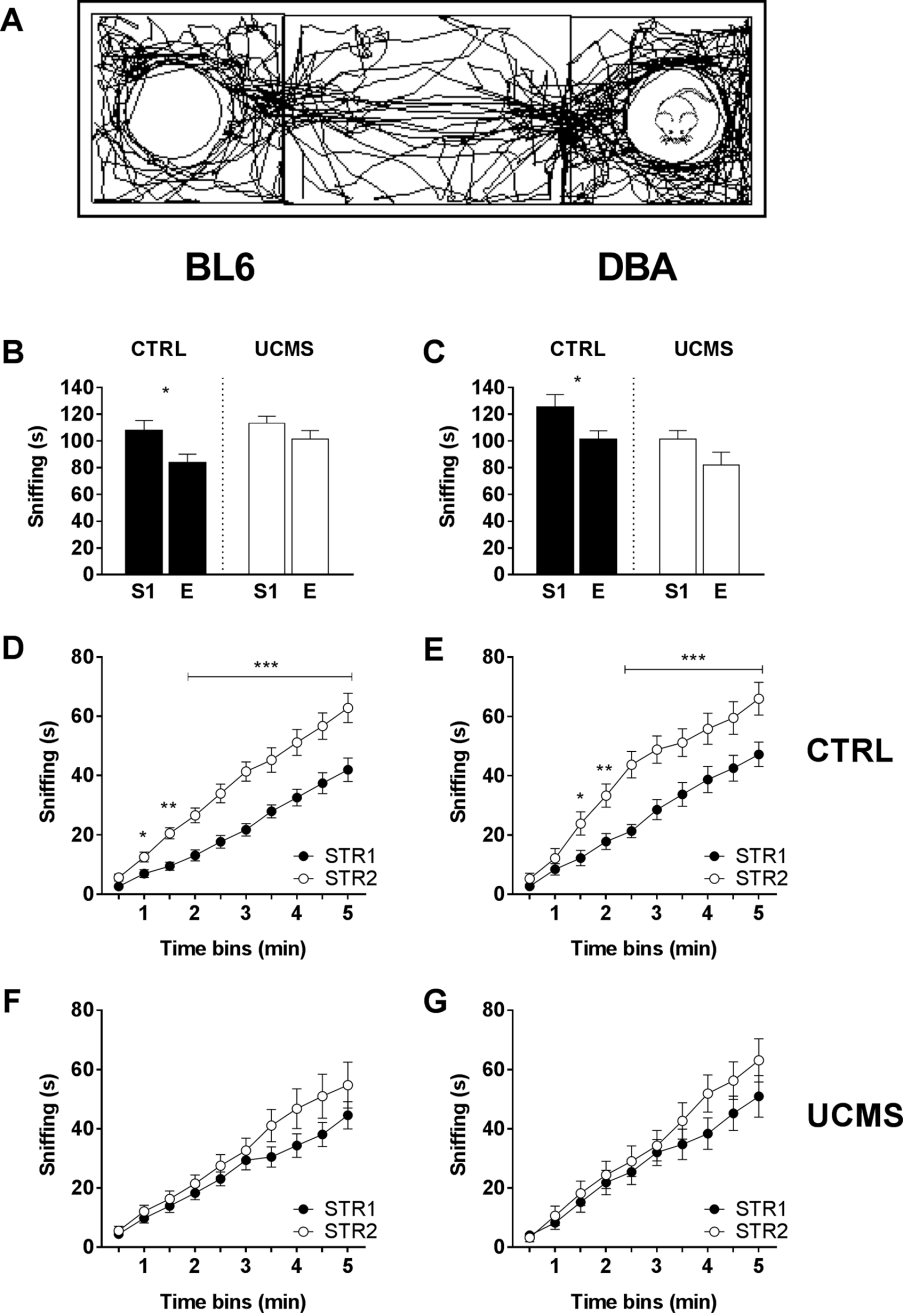
Sociability and preference for social novelty (SPSN). (A) Representative track plot of single sociability trial. (B) During sociability trials, BL6 CTRLS significantly preferred the STR1 (S1) chamber over the empty (E) chamber, whereas UCMS mice showed no preference. (C) Similar results were observed for DBA mice, where the UCMS group showed decreased sociability when compared to CTRLS. (D-F) During preference for social novelty trials, CTRL mice preferred the novel STR2 during social novelty exploration (first 5 min.). UCMS mice showed no clear preference for either STR1 or STR2. Values are expressed as mean +/- SEM. *, p < 0.05; ** p < 0.01; *** p < 0.001.

When presented with an unknown stranger mouse (Fig 3A), CTRL BL6 mice show increased sniffing behavior towards the stranger mouse, while UCMS BL6 are indifferent (for factor side: F(1, 28) = 8.662, p < 0.01; for factor condition: F(1, 28) = 4.775, p = 0.037; Fig 3B). Similarly, CTRL DBA show more interest towards a stranger mouse, while UCMS DBA are indifferent (for factor side: F(1, 28) = 5.534, p = 0.026; for factor condition: F(1, 28) = 9.92, p < 0.01; Fig 3C). These results suggest that stress decreases explorative sniffing behavior towards a stranger mouse and affects sociability in both strains similarly.

During preference for social novelty testing, CTRL animals showed a preference towards a novel stranger. When adding up the time animals spent sniffing either stranger mouse, both CTRL group show a clear increase in sniffing time towards STR2 compared to STR1 (BL6: F(1,14) = 15.15, p < 0.01; DBA: F(1,14) = 6.886 p = 0.02; Fig 3D and 3E). In contrast, exposure to UCMS reduced the preference for social novelty in both BL6 [F(1,14) = 1.323, p = 0.269; Fig 3F] and DBA [F(1,14) = 0.665 p = 0.428; Fig 3G]. Taken together these results suggest similar behavioral effects of UCMS exposure across strains. Both UCMS-treated BL6 mice and UCMS-treated DBA mice show less sociability behavior. Moreover, both UCMS-treated BL6 mice and UCMS-treated DBA mice failed to (socially) discriminate between a novel and familiar stranger mouse.

### Contextual threat conditioning and discrimination

#### Context threat acquisition

**Fig 4:**
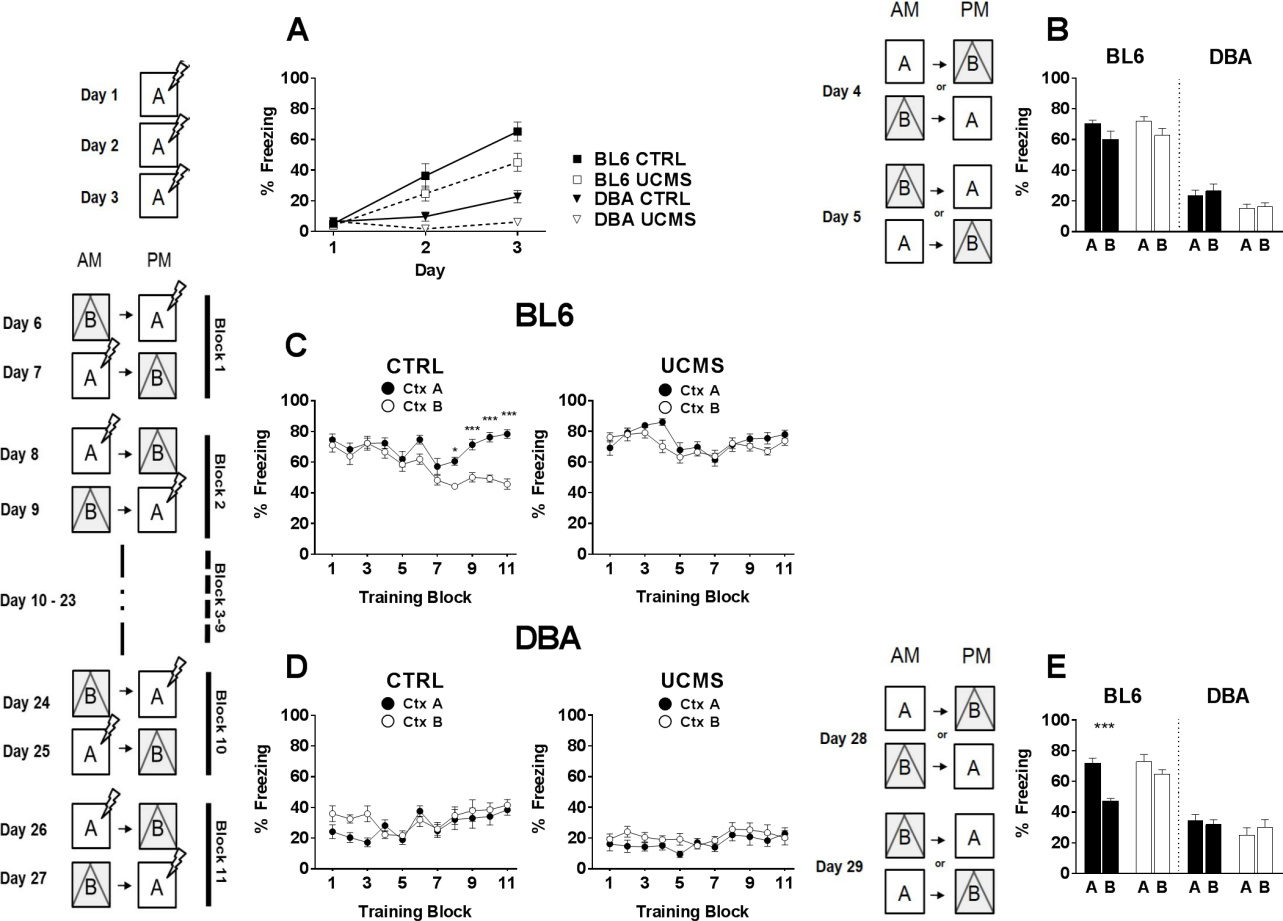
Contextual discrimination threat conditioning. (A) During context acquisition conditioning, mice exposed to UCMS showed less freezing behavior over time when compared to non-exposed controls. (B) Equivalent freezing behavior between UCMS-exposed and control groups was observed during generalization tests. (C) During context discrimination training, BL6 CTRL mice learned to distinguish context A from context B, whereas UCMS exposed mice did not. (D) DBA mice overall showed little freezing behavior and failed to distinguish context A from B, regardless of UCMS exposure. (E) A second generalization test revealed increased contextual discrimination behavior in unexposed BL6 controls. Values are expressed as mean +/- SEM. *, p < 0.05; ** p < 0.01; *** p < 0.001.

Contextual threat conditioning induced freezing behavior in all mice (Fig 4A). RM-ANOVA indicated a main effect of within-subjects factor acquisition [F(1,44) = 63.91, p < 0.001]. However, main effects of between-subjects factors strain [F(1,44) = 50.52, p < 0.001] and UCMS-exposure [F(1,44) = 9.223, p < 0.01] suggest that groups did not freeze similarly. We observed that BL6 animals in general displayed more robust freezing behavior than DBA. This difference has been reported before [62,63] and has been linked to differences in persistence of hippocampal long-term potentiation [53]. In both strains, UCMS animals displayed reduced freezing behavior over 3 days of threat conditioning (BL6: F(1,22) = 4.628, p = 0.043; DBA: F(1,22) = 9.424, p < 0.01).

#### Generalization test

Fear generalization towards different contexts was tested on days 4 and 5 (Fig 4B). Animals were exposed to two highly similar contexts to evaluate fear generalization in the absence of shocks. In the BL6 strain, two-way ANOVA indicated an effect of context [F(1,44) = 5.663, p = 0.021], but not of condition [F(1,44) = 0.341, p < 0.001]. In the DBA strain, two-way ANOVA indicated an effect of condition [F(1,44) = 7.534, p < 0.01], but not of context [F(1,44) = 0.337, p = 0.564].

#### Discrimination training

Following the generalization test, mice were trained to discriminate between context A and context B for 22 consecutive days. In context A, mice received a shock after 3 min, while context B was without shock. Freezing in either context during the initial 3 min was plotted. CTRL BL6 learned to discriminate between contexts (RM ANOVA for freezing over blocks [F(10,220) = 4.494, p < 0.001]; for context [F(1,22) = 30.56, p < 0.001]; Fig 4C). In contrast, UCMS BL6 were unable to learn to discriminate and displayed consistent levels of freezing throughout the 22 days (RM ANOVA for freezing over blocks [F(10,220) = 1.701, p = 0.081]; for context [F(1,22) = 1.969, p = 0.174]; Fig 4C). Furthermore, CTRL DBA (Fig 4D) showed increased freezing over time [F(10,220) = 5.214, p = 0.040], but were unable to learn to discriminate [F(1,22) = 1.375, p = 0.253]. UCMS DBA (Fig 4D) overall showed very little freezing [F(10,220) = 0.875, p = 0.556] and never displayed context discrimination [F(1,22) = 1.429, p = 0.245].

#### Generalization test

Fear generalization between contexts was again tested on days 28 and 29 (Fig 4E). In the BL6 strain, two-way ANOVA indicated an effect of both context [F(1,44) = 22.26, p < 0.001] and condition [F(1,44) = 7.658, p < 0.01]. Post hoc analysis confirmed CTRL BL6 mice (p < 0.001) learned to discriminate between contexts, whereas UCMS BL6 mice (p = 0.499) failed to do so. DBA CTRL and UCMS mice showed similar behavioral patterns (two-way ANOVA for context [F(1,44) = 0.075, p = 0.786]; for condition [F(1,44) = 1.782, p = 0.189]). CTRL and UCMS mice failed to discriminate between contexts.

#### Biomarkers

In order to minimize circadian fluctuation, basal corticosterone concentrations were collected in the morning when circulating corticosterone levels are low [64]. Efforts were made to collect samples under stress-free conditions. In week 10, CORT analysis revealed no differences in concentrations of plasma corticosterone levels following exposure to UCMS in BL6 mice (Mann-Whitney test, p = 0.10) and DBA mice (Mann-Whitney test, p > 0.99) when compared to controls (see Table 2). CORT analysis conducted in week 17 revealed significantly higher plasma corticosterone levels in UCMS BL6 when compared to CTRL BL6 (Mann-Whitney test, *p = 0.016; Table 2). UCMS DBA mice did not display significantly higher concentrations of plasma corticosterone levels in comparison to CTRL DBA (Mann-Whitney test, p = 0.547; Table 2).

**Table 2:** Summary of blood plasma corticosterone quantification.

		*Avg*. *blood plasma CORT levels* *(ng/ml) in week 10 (n = 3/group)*	*Avg*. *blood plasma CORT levels (ng/ml) in week 17 (n = 5/group)*
**BL6**	Control	348 ± 15.2	247 ± 45.7
	UCMS	657 ± 258.2	323 ± 43.3*
**DBA**	Control	320 ± 136.7	289 ± 143.5
	UCMS	457 ± 124.8	354 ± 190

UCMS increased significantly corticosterone levels in blood plasma (Mann-Whitney test * P<0.05)

**Fig 5:**
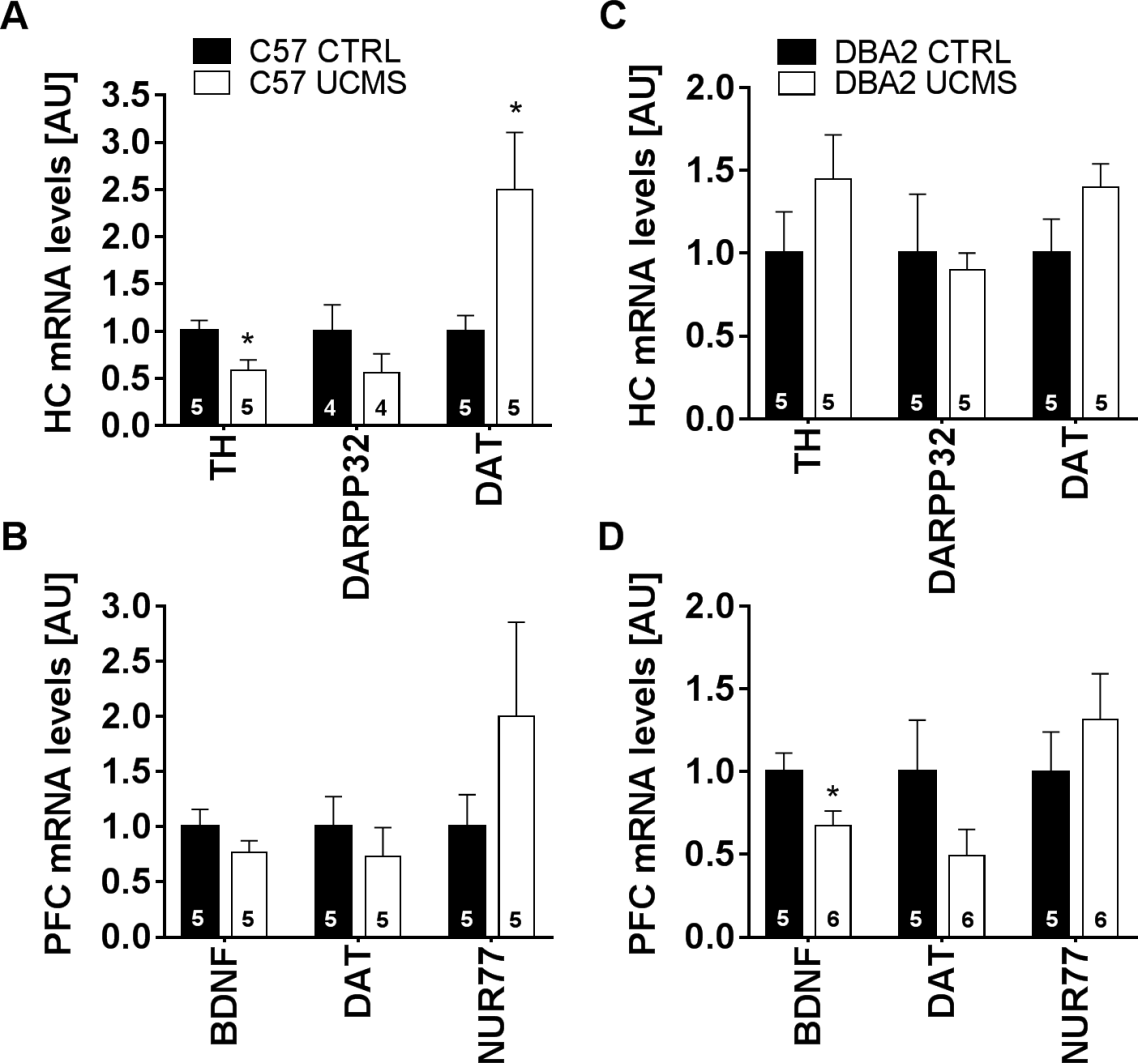
qRT-PCR assays. (A) Hippocampal gene expression analysis revealed significantly decreased TH mRNA levels (n = 5) and DAT (n = 5) mRNA levels of UCMS exposed BL6 mice, relative to unexposed controls. (C) No such differences were observed between DBA groups. (B) PFC gene expression analysis of BL6 groups. (D) PFC gene expression analysis revealed decreased BDNF (n = 5) mRNA levels in DBA2 mice, relative to unexposed controls (n = 6). Values are expressed as mean +/- SEM. *, p < 0.05; ** p < 0.01; *** p < 0.001.

Furthermore, qRT-PCR analysis was performed to check for hippocampal and prefrontal cortical changes in expression of genes involved in dopaminergic neurotransmission and neuronal plasticity. Differences in hippocampal expression of genes related to dopaminergic neurotransmission were observed between BL6 groups, but not between DBA groups (Fig 5B and 5C). In terms of altered dopaminergic neurotransmission, UCMS BL6 mice showed significantly decreased tyrosine hydroxylase (TH) mRNA levels (Mann-Whitney test, *p = 0.027) and significantly increased dopamine transporter (DAT) mRNA levels (Mann-Whitney test, *p = 0.045) when compared to CTRL. Moreover, analysis of PFC gene expression (Fig 5B and 5C) showed significantly decreased Brain-Derived Neurotrophic Factor (BDNF) mRNA levels in UCMS DBA mice relative to unexposed CTRL (Mann-Whitney test, *p = 0.030). Expression levels of other genes related to dopaminergic signaling (such as D1R, D2R, COMT and VMAT2) were assessed in HC and PFC, with no differences observed across groups (data not shown).

## Discussion

In this study we compared the effect of chronic mild stress on exploration, social and cognitive behavior in two inbred mouse strains, C57BL/6j and DBA2 (referred to as BL6 and DBA, respectively). We observed that UCMS had a similar effect on exploratory and social behavior in both mouse strains, but distinct effects on discrimination learning in contextual threat conditioning. Furthermore, we found a differential effect on genes associated with dopaminergic neurotransmission in the two mouse strains.

BL6 and DBA groups have been shown to have strain-specific activity patterns in the overnight activity test [65], and in exploration and anxiety tests [66]. We observe that upon exposure to UCMS both BL6 and DBA mice strains showed comparable changes in behavioral patterns in these particular tests. Stress-exposed mice have been reported to exhibit reduced nocturnal activity and reduced anticipation to morning light phase onset [67]. Animals selected for high reactivity to stress have been shown to have lower activity levels during the dark period as well as a shift in peak activity [64]. Disturbances in circadian rhythm and sleep pattern are also associated with stress-induced deregulation of the HPA axis and the pathophysiology of stress-related disorders [25,68]. Therefore, the changes we observed are in accordance with other stress models and with clinical observations and validate our UCMS procedure.

Exploration in the open field as well as the elevated plus maze is considered to have conflict resolution aspects, such as center exploration in the open field and entering open arms in the elevated plus maze. We observed that exposure to UCMS increased the time spent in the center in the open field and increased the number of entries in the open arms of the elevated plus maze. We interpret the observed effects of increased center activity and open arm visits as due to changes in arousal and increased reactivity to novelty rather than reduced anxiety [69]. Results from other tests support this interpretation. We observed increased exploratory activity specifically in DBA in four independent tests: in the first light phase of the 24h activity test, in the open field, elevated plus maze as well as in the SPSN test, while BL6 showed hyperactivity in elevated plus maze and a trend in the open field and 24h activity. Similar increases in center time and open arm visits have been reported before [46,69], but other studies reported reductions [70–73]. We attribute these contradictory results as being caused by differences in housing condition, testing history and strain background, factors that have all been shown to influence exploration in open field and elevated plus maze [74].

In the current study, UCMS-exposed BL6 and UCMS-exposed DBA mice exhibited reduced sociability behavior, a behavior that has been associated with models for autism spectrum disorder [75], schizophrenia [11,76], anxiety [77] and depression [78]. This effect of chronic stress on social behavior in rodents has been reported before [79]. In addition, neuropeptides of the corticotrophin-releasing factor family that coordinate stress response have been shown to affect social behavior as well [80]. In addition to reduced sociability behavior we found that chronically stressed animals of both strains failed to (socially) discriminate between a novel and familiar mouse during the social novelty phase. These results are consistent with a report by Van Kooij et al. [81] showing that chronic restraint stress has detrimental effects on sociability and social memory in rodents. Social recognition memory consolidation depends on a functional network involving the PFC, HC, anterior cingulate, and amygdala [82–84]. Tanimizu and colleagues speculated that the PFC-HC network is crucial in social recognition and discrimination. Our observations indicate that in particular social discrimination (familiar versus novel mouse) is impaired when animals are exposed to chronic mild stress. This impairment in discrimination was also observed in contextual threat conditioning to conceptually very similar contexts. We observed that after initial threat conditioning to a specific context, BL6 discriminated between highly similar contexts, but UCMS had no effect on the discrimination. In contrast, DBA animals were unable to distinguish between the conditioned context and a highly similar context showing similar freezing responses to either context. This indicates that the contexts were similar enough to create a high generalization effect. Overall, we observed that the freezing level was much higher in BL6 than in DBA, however, this might not per se reflect memory impairment, but simply an increased activity level in DBA. Indeed, freezing levels in DBA were higher after conditioning, indicating that DBA are able to form an aversive contextual representation.

During discrimination training, animals are repeatedly exposed to the aversive and the neutral context and their behavior becomes over time more context specific. This process of discriminative learning has been shown to depend on a functional PFC-HC network [85,86]. The interplay of both PFC and HC is crucial in a series of adaptive learning paradigms such as reversal learning, threat extinction, context discrimination and generalization [87,88]. Stress-related disorders such as MDD have been linked to reduced mnemonic discrimination performance [89]. It has been argued that this propensity to form or recall information in an unspecific, more generalized way, could in part be due to reduced hippocampal function and connectivity [90]. UCMS mice were expected to perform worse in this complex cognitive task. Indeed, we confirmed that UCMS-exposure significantly diminished discrimination ability in BL6 mice compared to controls. These results are consistent with previous findings showing that chronic stress significantly impairs cognitive performance in discriminative fear conditioning [91], novel object recognition [44] and spatial memory [17,92,93]. However, our data indicate that these effects are strain-specific. Despite extensive conditioning, DBA mice exerted less context-evoked freezing behaviour when compared to BL6 mice. Moreover, neither UCMS-exposed DBA mice nor control DBA mice could successfully discriminate between contexts. When compared to BL6 mice of the standard genetic background, mice of the genetically unrelated DBA strain typically show more resilience to helplessness induced by an unavoidable stressor [94]. For example, exposure to an inescapable shock has been shown to reduce activity in a Y maze in BL6 mice, but not in DBA mice [95]. A possible explanation might be that DBA mice have altered HC-PFC functioning and therefore perform poorly on learning and memory tasks, and that chronic stress has very little influence on cognitive performance. Studies have shown DBA mice to have reduced persistence of hippocampal long-term potentiation (LTP) when compared to BL6 mice, as well as reduced levels of signalling proteins such as protein kinase C (PKC) [63,96]. Furthermore, DBA mice exert reduced levels of cAMP response element-binding protein (CREB), a transcription factor associated with long-term memory formation, in prefrontal cortex and hippocampus when compared to BL6 mice [97]. These differences manifest themselves in cognitive performances where strain-dependent differences have been observed in various spatial memory and contextual conditioning tasks. Specifically, DBA mice are outperformed by BL6 mice in Barnes maze [98], Morris water maze [99] and contextual fear conditioning [63]. Together, our data suggest that chronic exposure to stress could lead to impairments in HC-dependent social and cognitive behavior, comparable to what is seen in various stress-related psychopathologies [78,90,100,101].

Across the many gene sets involved in these divergent behavioural responses to chronic stress, we chose to focus on genes involved in neural plasticity and dopaminergic neurotransmission in HC and PFC. In addition to its role in anhedonia, motivation and the regulation of circadian rhythm, dopamine might play an important role in stress-induced social and cognitive impairments [43,102,103]. Specifically, stress-induced disturbances in neural plasticity have been associated with a hypodopaminergic state, which leads to disturbances in neural plasticity persistence [23,104]. We quantified the expression of tyrosine hydroxylase which catalyses the L-tyrosine to L-DOPA conversion. In the hippocampus of BL6 mice, UCMS induced a significant downregulation of TH expression. We also measured a downregulation of DARPP32, a post-synaptic inhibitor of PPP1CA, which has been linked to learning and memory defects [105,106]. DARPP32 is activated upon D1R activation [107], and a downregulation of this gene is suggestive of post-synaptic DAergic signal modulation. In addition, we observed an upregulation of presynaptic DA transporter (DAT) in response to UCMS in the HC of BL6. The upregulation of DAT, together with reduction in TH might reflect reduced hippocampal availability of DA as it has been shown that an increase in DA reuptake can induce a reduction of DA catalysis by TH in DA neurons [108]. Reduction in DA neuromodulation in HC has been linked to impaired learning and memory, in particular pattern separation [109]. Unexpectedly, UCMS did not affect TH, DARPP32, nor DAT in the HC of DBA mice.

Nevertheless, DBA mice have reportedly reduced responsiveness to DA releasing drugs such as cocaine and d-amphetamine [110–112], and altered D1/D2 receptor balance in the hippocampus [113]. Thus, the observed differences might be due to the involvement of stress-induced expression changes of different genes in the brain of DBA and BL6 mice [50], as DBA and BL6 mice show opposite differences in brain dopamine functioning under stressful conditions [114]. Specifically, DBA animals have an increased number of neurons positive for dopamine transporter (DAT) and tyrosine hydroxylase (TH) in HC and PFC [113,115]. These different DA systems might be differentially involved in liability to chronic stress and the observed social and cognitive impairments, underlining the importance of combining different inbred strains with different behavioral test batteries to study gene-environment interactions involved in the pathological outcomes of stress exposure [116].

In PFC, expression of transcription factors (NUR77) involved in the development and differentiation of dopamine neurons was similarly, but insignificantly, increased across strains [117]. Furthermore, the neurotrophin family member BDNF was significantly downregulated in DBA, not in BL6 mice. BL6 mice did show a similar, albeit not significant, expression pattern. Although inconsistencies in UCMS expression data have been reported, the lack of robust down-regulation of classic biomarkers for stress-induced neural dysfunctions may be related to the procedural design used in the current study [118,119]. Both UCMS and control mice were repeatedly exposed to mild foot shocks during discrimination training, a regimen comparable to the chronic stress procedure itself. Exposure to a single acute stressor has been shown to be sufficient for altered expression levels of the investigated cellular targets involved in learning and memory [120–122]. Therefore, due to similar stress-induced changes in gene expression levels of control animals, changes related to the UCMS-procedure itself might not have been apparent.

Taken together, our findings indicate that a chronic mild stress regimen lead to explorative, social and cognitive impairments. Stress-induced cognitive impairments could be related to altered dopaminergic neurotransmission in Bl6 mice, but not in DBA mice. We propose that applying UCMS to females of different inbred strains is a good model to study the impact of a genetic background and environmental factors in humans where some individuals show more susceptibility to developing stress-related disorder then others. Our results also highlight the importance of studying different strains in multiple test batteries, to help researchers in choosing appropriate strains for the analysis of the neural and genetic basis of stress-induced impairments in the anxiety, social and cognitive domain.
